# Advantages of cone beam computed tomography for evaluation of subchondral insufficiency fractures of the knee compared to MRI

**DOI:** 10.1038/s41598-024-64591-7

**Published:** 2024-07-03

**Authors:** Maximilian M. Delsmann, Julian Delsmann, Nico Maximilian Jandl, Kai-Jonathan Maas, Frank Timo Beil, Michael Amling, Frank Oliver Henes, Tim Rolvien, Clemens Spink

**Affiliations:** 1 Department of Osteology and Biomechanics, University Medical Center Hamburg-Eppendorf, Lottestraße 59, 22529 Hamburg, Germany; 2 Department of Trauma and Orthopaedic Surgery, Division of Orthopaedics, University Medical Center Hamburg-Eppendorf, Martinistraße 52, 20246 Hamburg, Germany; 3 Department of Diagnostic and Interventional Radiology and Nuclear Medicine, University Medical Center Hamburg-Eppendorf, Martinistraße 52, 20246 Hamburg, Germany; 4 Department of Diagnostic and Interventional Radiology, BG Hospital Hamburg, Hamburg, Germany

**Keywords:** Subchondral bone plate, Collapse, Osteonecrosis, SPONK, Ahlbäck, Insufficiency fracture, SIFK, CBCT, Medical research, Trauma

## Abstract

To determine the diagnostic yield of cone beam computed tomography (CBCT) compared with 3 T magnetic resonance imaging (MRI) for the evaluation of subchondral insufficiency fractures of the knee. Consecutive patients with subchondral insufficiency fractures of the knee examined by 3 T MRI and CBCT of the femoral condyles were reviewed. Two experienced raters graded the lesion severity on 3 T MRI and CBCT images: grade 1: no signs of a subchondral bone lesion; grade 2: subchondral trabecular fracture or cystic changes, but without infraction of the subchondral bone plate; grade 3: collapse of the subchondral bone plate. Ratings were repeated after six weeks to determine reliability. In addition, the bone lesion size was measured as elliptical area (mm^2^) and compared between CBCT and T1-weighted MRI sequences. Among 30 patients included (43.3% women; mean age: 60.9 ± 12.8 years; body mass index (BMI) 29.0 ± 12.8 kg/m^2^), the medial femoral condyle was affected in 21/30 patients (70%). The grading of subchondral lesions between MRI and CBCT did not match in 12 cases (40%). Based on MRI images, an underestimation (i.e., undergrading) compared with CBCT was observed in nine cases (30%), whereas overgrading occurred in three cases (10%). Compared to CBCT, routine T1-weighted 3 T sequences significantly overestimated osseus defect zones in sagittal (84.7 ± 68.9 mm^2^ vs. 35.9 ± 38.2 mm^2^, p < 0.01, Cohen’s *d* = 1.14) and coronal orientation (53.1 ± 24.0 mm^2^ vs*.* 22.0 ± 15.2 mm^2^, p < 0.01, Cohen’s *d* = 1.23). The reproducibility of the grading determined by intra- and inter-rater agreement was very high in MRI (intra-class correlation coefficient (ICC) 0.78 and 0.90, respectively) and CBCT (ICC 0.96 and 0.96, respectively). In patients with subchondral insufficiency fractures of the knee, the use of CBCT revealed discrepancies in lesion grading compared with MRI. These findings are clinically relevant, as precise determination of subchondral bone plate integrity may influence the decision about conservative or surgical treatment. CBCT represents our imaging modality of choice for grading the lesion and assessing subchondral bone plate integrity. MRI remains the gold standard modality to detect especially early stages.

## Introduction

Subchondral insufficiency fracture of the knee, also known as spontaneous osteonecrosis of the knee (SONK) or Morbus Ahlbäck, is a musculoskeletal condition that usually presents clinically as atraumatic, sudden, nonspecific knee pain accompanied by bone marrow edema (BME)^1–3^. This lesion typically occurs in the medial femoral condyle and is most common in patients older than 50 years of age^4–6^. While the nomenclature and assumed pathogenesis vary in the literature, subchondral insufficiency fractures appear to initially develop from BME (also termed bone marrow lesion) with an increased risk of secondary osseous lesions and subchondral bone plate instability along progression^3,7^. Consequently, they are a well-known factor contributing to the development of knee osteoarthritis (OA)^8^.

The continuity of the subchondral bone plate is of decisive importance for defining the optimal treatment algorithm. Whereas in cases of an intact subchondral bone plate, conservative treatment represents a probate option, surgical evaluation is usually recommended in case of a collapsed subchondral bone plate^2,9–12^. Conservative treatment usually includes reduced weight bearing, optimized supplementation with vitamin D and calcium as well as antiresorptive therapy with denosumab or bisphosphonates^9–12^. Surgical procedures include subchondral core decompression, osteochondral grafting, autologous chondrocyte implantation or even total-/uni-compartmental knee arthroplasty^13–17^.

For subchondral insufficiency fracture grading and osseous defect size evaluation, different imaging techniques are available. Multi-slice imaging techniques of magnetic resonance imaging (MRI) as the gold standard modality and occasionally additional multidetector computed tomography (MDCT) are preferably used for subchondral insufficiency fracture evaluation and therapeutic decision-making. Proton density (PD) MRI sequences provide a high diagnostic accuracy in detecting early stages of lesions such as BME without osseous defects and T1-weighted sequences allow a detection of osseous lesions albeit with less diagnostic accuracy than computed tomography^3,18^.

Cone beam computed tomography (CBCT), first utilized in maxillofacial surgery and dentistry, is a promising imaging technique using a three-dimensional conical X-ray beam in combination with a flat panel detector^19^. By providing higher sensitivity and specificity in detecting fractures compared to X-ray, as well as higher spatial resolution and lower radiation exposure than MDCT, CBCT is of growing interest for extremity imaging in traumatology and orthopedics^20,21^.

The aim of this retrospective study was to investigate the diagnostic yield of CBCT in comparison with 3 T MRI in the evaluation of subchondral insufficiency fractures the femoral condyle.

## Materials and methods

### Patients & study design

We retrospectively analyzed 30 Caucasian consecutive patients who presented at our outpatient clinic with new-onset atraumatic knee pain and no history of OA. MRI had been performed prior to presentation, showing femoral BME with suspected subchondral insufficiency fracture. CBCT of the knee was performed as part of the clinical routine to additionally evaluate the integrity of the subchondral bone plate to derive recommendations for conservative vs. surgical treatment options. In all patients, the time interval between MRI and CBCT was no longer than three months. None of the following criteria were present in our patients: History of an inciting trauma or infection of the affected knee, hereditary or metabolic diseases (e.g. hypophosphatasia), cancer or treatment-induced bone loss. Thus, no patient had to be excluded. This study was performed in accordance with the local ethics committee and was approved by the institutional review board. Informed written consent was obtained from all patients. All investigations were performed in accordance with the Declaration of Helsinki.

### MRI and CBCT imaging protocols

The outpatient MRI datasets were performed on 3 T-scanners of different vendors. The routine imaging protocol consisted of a 3D PD weighted fat-saturated sequences in sagittal orientation with axial and coronal reformations (mean TR 1305.6 ± 157.4 ms; mean TE 37.2 ± 3.6 ms, FoV 180 × 180 mm; matrix 320 × 320; slice thickness: 3 mm) and T1w turbo spin echo (TSE) sequences in coronal and sagittal orientation (mean TR 818.1 ± 75.4 ms; mean TE 10.8 ± 1.9 ms; FoV 180 × 180 mm; matrix 672 × 672; slice thickness: 2 mm).

For CBCT imaging (SCS MedSeries^®^ H22, CE Planmed Verity, Helsinky, Finland) the following routine settings were used: tube voltage 92 kV, tube current of 5 mA with a slice thickness of 0.2 mm, rotation time 23 s (pulsed, effective) on a FOV of 16 × 8.5 cm. The tube rotation angle was 210°. The dose length product (DLP) during CBCT imaging of the knee joint was in all cases 705 mGy × cm^2^.

### Imaging analysis

**Figure 1:**
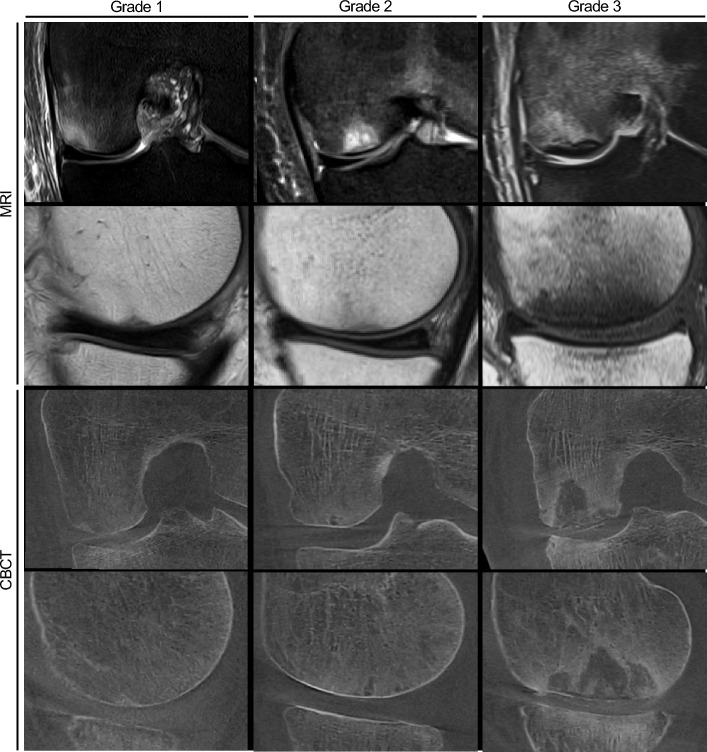
Grading of subchondral insufficiency fractures in MRI vs. CBCT. *MRI* magnetic resonance imaging; *CBCT* cone beam computed tomography. Grade 1: no visible subchondral bony lesion in MRI or CBCT. Bone marrow edema in the femoral condyle with elevated signal in PD (first row) and decreased signal in T1 MRI sequences (second row). Grade 2: subchondral fracture or cystic changes with circumscribed lesions of highly elevated signal in PD sequences without an infraction of the subchondral bone plate in CBCT. Grade 3: subchondral bone plate collapse and with corresponding cortical discontinuity of the cortical delimitation in T1 sequences and circumscribed overlying articular surface step offs in CBCT.

Two radiologists (C.S. and K.-J.M) with 7- and 5 year’s experience in musculoskeletal imaging compared the subchondral insufficiency fracture grading and defect size in MRI and CBCT, using a modified classification^3^ (Fig. 1):

- Grade 1: no visible subchondral bony lesion
- Grade 2: subchondral fracture or cystic changes without an infraction of the subchondral bone plate
- Grade 3: subchondral bone plate collapse and depression of the articular surface with a step off.

Measurement of subchondral lesion size was performed in coronal and sagittal orientations in T1-weighted MRI sequences and CBCT by calculating an elliptical area. Following formula was used: height (mm) × depth (or width; mm) × π = area (mm^2^). Free windowing during analyses in CBCT was allowed. Both readers repeated the grading and measurements after a 6 week interval to evaluate intra- and inter-rater reliability. Due to the high level of agreement, data from the more experienced rater at the first evaluation time point (C.S.) were used for further analysis.

### Dual-energy X-ray absorptiometry (DXA)

As part of the skeletal status assessment, we measured the bone mineral density (BMD) in the left and right proximal femur (total hip) and in the lumbar spine (L1–L4) in all patients using dual-energy X-ray absorptiometry (DXA; Lunar iDXA, GE Healthcare; Madison, WI, USA) according to the German osteoporosis guidelines (DVO). T-scores representing BMD standard deviations for young, sex-matched healthy adults were generated using the manufacturer’s software. Osteoporosis and osteopenia were diagnosed based on T-scores according to World Health Organization (WHO) guidelines (i.e., normal T-score >  − 1.0, osteopenia T-score >  − 2.5 ≤  − 1.0, osteoporosis T-score ≤  − 2.5).

### Statistical analysis

Statistical analysis was performed by using GraphPad Prism^®^ (version 9.0, GraphPad Software, La Jolla, CA) and SPSS^®^ statistical program (version 26.0, IBM, Armonk, New York, USA). Continuous variables are expressed as absolute values or the mean ± standard deviation (SD). Shapiro–Wilk test was performed to evaluate the normal distribution of the data. We used student’s *t*-test for normally distributed data and Mann–Whitney *U*-test for non-normally distributed data. In order to assess the level of agreement between inter-modality gradings, Cohen’s Kappa was calculated. Kappa values range from 0, indicating no agreement, to 1.00, indicating almost perfect agreement. The evaluation was carried out using the interpretation guidelines. Cohen’s Κappa of ≥ 0.2 indicates a fair, ≥ 0.4 a moderate, ≥ 0.6 a good and ≥ 0.8 a very good agreement^22^. Furthermore, the effect size Cohen’s *d* was calculated to determine the statistical power of lesion size analysis. Values of *d* ≥ 0.20 represent a small effect, *d* ≥ 0.50 a medium effect and values from *d* ≥ 0.80 a large effect^23^. Intraclass correlation coefficient (ICC, absolute agreement, two-sided mixed effects, multiple measures) was calculated to determine the agreement between the two rater’s evaluations at the two timepoints by intra- and inter-rater reliability. Based on 95% confident intervals (CIs), ICCs indicated poor (< 0.5), moderate (0.5–0.75), good (0.75–0.9) and excellent (> 0.9) reliability^24^. Statistical significance was set to a two-tailed p-value of < 0.05.

### Ethical approval

This study was performed in accordance with the local ethics committee and was approved by the institutional review board (2021-300069-WF).

### Informed consent

The authors declare that informed consent was obtained in all patients.

## Results

The study cohort of 30 Caucasian patients consisted of 13 women (43.3%) with a mean age of 60.9 ± 12.8 years. No age differences could be found between the sexes (women 64.71 ± 13.9 years vs. men 59.31 ± 14.22 years, p = 0.22). Basic demographic characteristics and the time intervals from MRI to CBCT between the sexes are shown in Table 1. The medial femoral condyle was affected in 21/30 patients (70%). With a mean BMI of 29.0 ± 12.8 kg/m^2^, the major proportion of the patients included in this study were categorized as obese according to WHO criteria (female: 29.31 ± 10.24 vs. men: 28.79 ± 3.57, p = 0.2; Table 1)^25^. Of the patient cohort, 9/30 patients (30.0%) suffered at least one fracture in their history, with vertebral fractures in 5/30 (16.6%) and peripheral fractures in five patients (5/30%). Evaluation of the DXA results showed that seven patients (23.3%) had normal T-scores, while the majority (n = 23; 76.7%) had reduced BMD (Table 1). Specifically, nine (30.0%) patients were found to have osteopenia and 14 patients (46.7%) were found to have osteoporosis. Prior to initial presentation to our department, seven patients received specific anti-osteoporotic treatment (23.3%).

**Table 1:** Demographics, time interval between MRI and CBCT, and skeletal status in female and male patients.

	Women n = 13	Men n = 17	p
Patient characteristics			
Age (years)	64.7 ± 13.9	59.3 ± 14.2	0.22
BMI (kg/m^2^)	29.3 ± 10.2	28.7 ± 3.5	0.20
MRI-CBCT time interval (days)	18.5 ± 14.3	37.1 ± 30.9	0.19
Previous fragility fractures			
Vertebral fractures	2/13	3/17	0.87^#^
Peripheral fractures	3/13	2/17	0.41^#^
DXA results			
Osteoporosis	10/13	4/17	**0.003**^**#**^
Osteopenia	2/13	7/17	0.13^#^
Specific anti-osteoporotic therapy	5/13	2/17	0.09^#^

Significant values are given in bold.

Results are presented as mean ± SD or n (%).

*BMI* body mass index, *MRI* magnetic resonance imaging, *CBCT* cone beam computed tomography, *DXA* dual-energy X-ray absorptiometry.

^#^determined by the Chi^2^ test.

The mean time interval between MRI and CBCT imaging was 29.4 ± 26.8 days (female: 18.5 ± 14.3 vs. men: 37.1 ± 31.0, p = 0.19; Table 1). After analysis of available MRI and CBCT imaging, findings were allocated to subchondral insufficiency fracture grades by the two experienced radiologists (Fig. 1). Agreement between the two raters (C.S. and K.-J.M), as well as between the two time points measured by inter- and intra-rater reliability, was good in MRI and excellent in CBCT (Table 2).

**Table 2:** Intra- and inter-rater agreement of subchondral fracture grading by intraclass correlation coefficient (ICC).

MRI		CBCT	
Intra-rater agreement (95% CI)	Inter-rater agreement (95% CI)	Intra-rater agreement (95% CI)	Inter-rater agreement (95% CI)
0.780 (0.633, 0.868)	0.901 (0.835, 0.941)	0.959 (0.932, 0.976)	0.959 (0.932, 0.976)

*MRI* magnetic resonance imaging, *CBCT* cone beam computed tomography, *CI* confidence interval.

**Figure 2:**
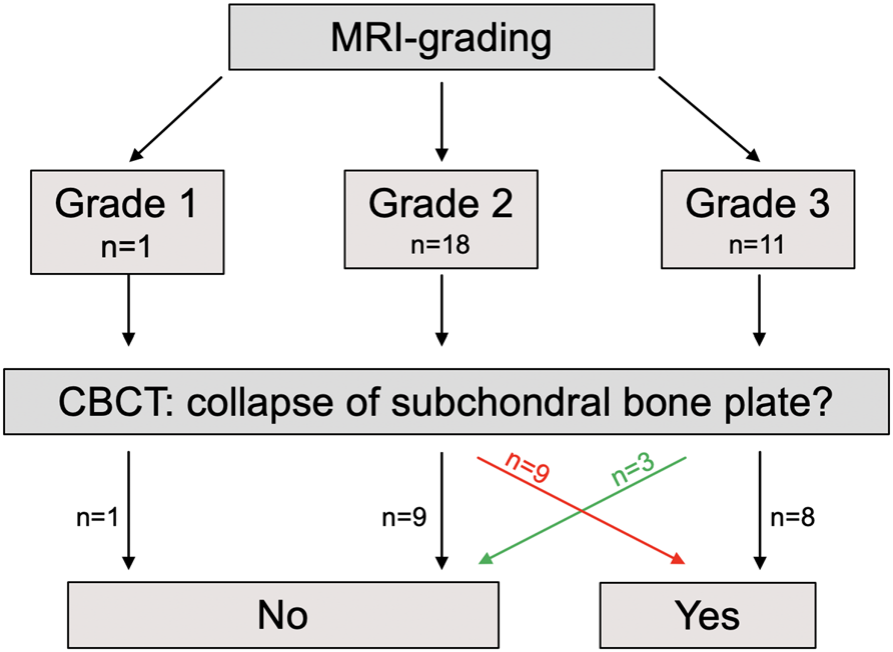
Distribution patterns and changes in subchondral insufficiency fracture (SIFK) grading on MRI followed by CBCT. *MRI* magnetic resonance imaging, *CBCT* cone beam computed tomography, *n* number of patients.

During analysis, it became evident that a considerable number of cases exhibited a discrepancy between the gradings based on MRI and CBCT (Fig. 2). More specifically, nine patients (30%) were diagnosed with higher lesion grading in CBCT, whereas three patients (10%) presented a milder grading. The most frequent reason for a discrepancy between the two modalities was a discrepancy in interpretation of the subchondral bone plate integrity (10/12 patients; 83.3%). The obtained Kappa value was 0.274 (95% CI − 0.01–0.56). In all patients with a higher CBCT-grading compared with MRI, the time interval between the two imaging methods were less than 1 month, and in 5/9 patients (55%) the interval was even less than 10 days.

**Figure 3:**
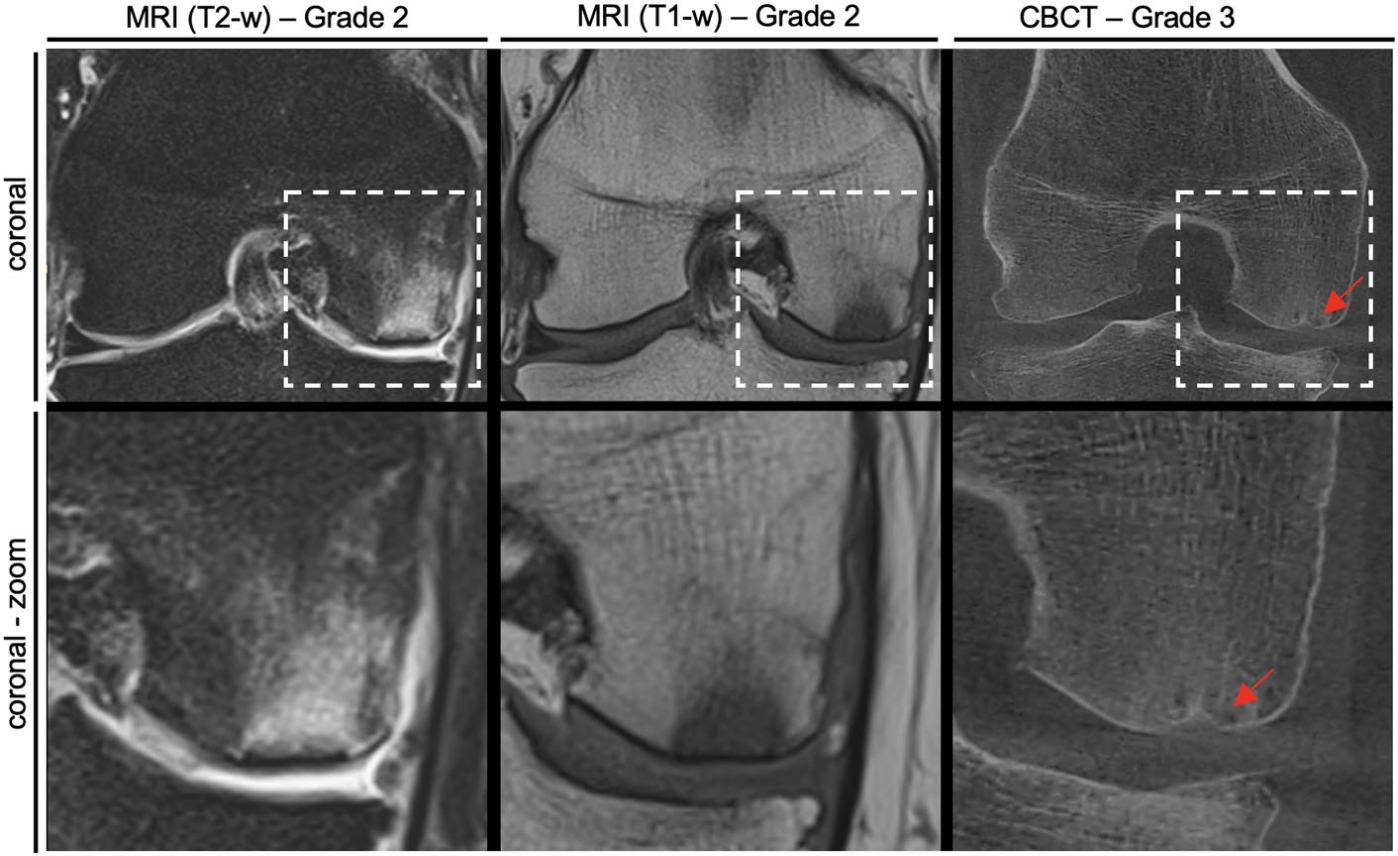
Representative case of a 61 year-old man with differing subchondral fracture gradings on MRI and CBCT. *MRI* magnetic resonance imaging, *CBCT* cone beam computed tomography. Note the grade 2 MRI findings of diffuse bone marrow edema, suspected subchondral fracture and cortical irregularities but without a circumscribed osseous infraction of the subchondral bone plate. Finally, CBCT performed three days after MRI unmasks a cortical step off with an infraction of the subchondral bone plate, suggesting a subchondral fracture grade 3 lesion.

The example of a 61 year-old man with atraumatic knee pain showed diffuse BME with subchondral cystic changes of the medial femoral condyle, but an intact and continuous subchondral bone plate in MRI, and the lesion was therefore classified as grade 2 (Fig. 3). However, CBCT performed 3 days after the MRI showed a significant collapse of the subchondral bone plate. It was interpreted as grade 3, representing clinically relevant undergrading of the subchondral fractures on MRI sequences.

**Figure 4:**
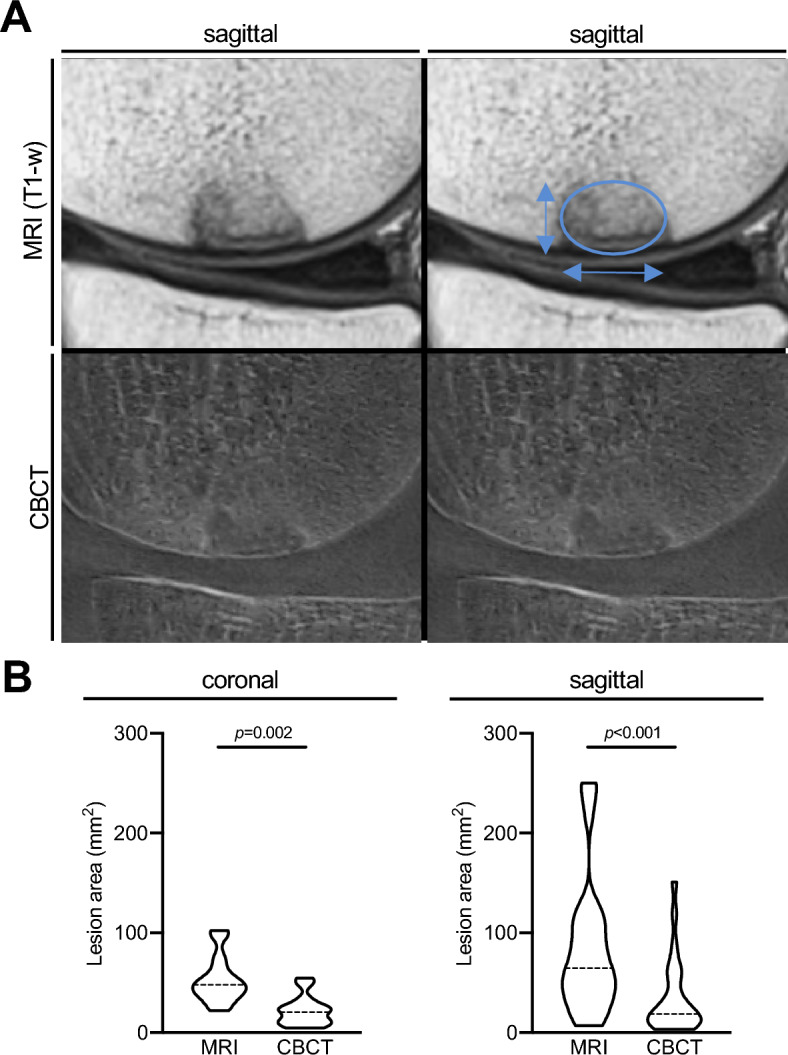
Measurement of the osseous lesion size using an elliptical area between MRI and CBCT. (**A**) Exemplary MRI (T1) and CBCT scans in sagittal orientation of a patient’s medial femoral condyle with measurement of the bony lesion. (**B**) Comparison of bony lesion size measured in coronal and sagittal views between MRI and CBCT. *MRI* magnetic resonance imaging, *CBCT* cone beam computed tomography, *T1-w* T1-weighted.

Analyzing the subchondral bony lesion sizes in T1-weighted MRI sequences and CBCT (Fig. 4A), the area interpreted as osseous defect zone was markedly lower in CBCT compared to MRI in both sagittal (35.9 ± 38.2 mm^2^ vs*.* 84.7 ± 68.9 mm^2^, p < 0.001, Cohen’s *d* = 1.14, 95% CI 0.62–1.65) and coronal projections (22.0 ± 15.2 mm^2^ vs. 53.1 ± 24.0 mm^2^, p < 0.001, Cohen’s *d* = 1.23, 95% CI 0.51–1.92; Fig. 4B).

## Discussion

In the present study, we investigated the diagnostic yield of CBCT compared with MRI in the evaluation of subchondral insufficiency fractures of the knee, a common cause of secondary OA and knee arthroplasty^8,15^. Particular attention was paid to the continuity and integrity of the subchondral bone plate as well as to the osseous lesion size, which are clinically relevant in terms of treatment recommendation and outcome. Comparison of MRI and CBCT revealed discrepancies in 40% of cases, with CBCT offering advantages in lesion characterization and MRI frequently underestimating damage of the subchondral bone plate while overestimating lesion size.

An accurate assessment of the subchondral bone plate is of pivotal importance to predict the risk of secondary OA and to select the most appropriate treatment algorithm. In the presence of BME with an intact subchondral bone plate, common treatment algorithms consist of adjusted weight-bearing, vitamin D and calcium supplementation, and, in case of increased bone resorption parameters or clinical indications such as rapid progressive BME, additional antiresorptive agents. In this context, antiresorptive drugs like the monoclonal RANKL-antibody denosumab or intravenous bisphosphonates may be an effective and safe option to preserve the cortical and trabecular bone microarchitecture and to counteract progression of BME to osteonecrosis if surgical measures are not indicated^9–11,26^. In the case of an infracted subchondral bone plate, surgery is often recommended. Based on other factors such as anatomic location and lesions size, the most suitable surgical option is determined, such as core decompression, osteochondral grafting, autologous chondrocyte implantation or, in the case of severe findings, partial or total knee arthroplasty^13–17^. Furthermore, accurate verification of the bony lesion and therefore the subchondral bone plate is also important to evaluate possible progression.

In the present study, it became evident during image analysis that the subchondral insufficiency fracture grading in MRI and CBCT varied in a substantial proportion of patients. The key factor for the discrepancy in subchondral fracture grading between these two modalities was, in most cases, a difference in the assessment of the integrity and continuity of the subchondral bone plate. While the subchondral bone plate frequently seemed not to be clearly delineated in both T1- and PD-weighted MRI sequences, the continuity and integrity of these fine bony structure could be assessed more precisely on CBCT imaging. As a result, subchondral insufficiency fracture grading had to be upgraded after having performed CBCT. The kappa value of 0.274 emphasizes this by describing only a fair degree of agreement between the gradings of the two modalities, which implies a considerable amount of disagreement.

MRI appears to be inferior in assessing bony structures compared to CBCT due to the uniform BME pattern in PD and even in T1 sequences. Accurate identification of the bony defect zone can be essential for the evaluation of conservative options, but also for accurate surgical planning. As for the size of the osteochondral lesion, we demonstrated that the bony lesion areas were smaller on CBCT. This indicates that while the subchondral bone plate defect is underestimated, the lesion size is overestimated on MRI. Interestingly, CT previously also showed diagnostic advantages over MRI in characterizing primary and secondary changes in other osteochondral lesions such as capitellar osteochondrosis dissecans of the elbow^27^. In this previous study, it was shown that fragmentation of osteochondrosis dissecans and secondary changes could be diagnosed more frequently and more accurately on CT and that more precise indications for surgery could subsequently be made. Also, similar to our investigation, the osseous lesion area could be assessed more preciously in the X-ray based CT modality compared to MRI.

Limitations of this study include the inability to compare BME extent between MRI and CBCT and the lack of comparison between CBCT and other X-ray-based imaging techniques such as conventional X-ray and MDCT. It can be concluded that MRI is superior to CBCT in the assessment of early stages. Novel CT techniques such as dual-energy CT (DECT) also have the potential to detect and quantify BME based on the differential energy-dependent x-ray absorption behaviors^28,29^. Virtual non-calcium techniques can suppress the high attenuation of trabecular bone and enable detection of subtle changes in bone marrow constitution. These techniques are able to detect even the first grade of subchondral lesions by displaying the early BME. While not applied in this study, the possibilities of edema detection by CT modalities should be evaluated in more detail in future studies. Two studies have previously demonstrated the potential of CBCT in BME detection and water quantification in phantoms^30,31^.

A comparison between CBCT and MDCT was not performed due to radiation safety concerns. While routine MDCT scans and reconstructions of the knee in our department usually contain a slice thickness of 1–2 mm, CBCT with a slice thickness of 0.2 mm clearly provides superior properties. In addition, CBCT of the knee potentially reduces the effective radiation dose by more than 50% compared to MDCT^32^. Therefore, it can be stated that CBCT has a more favorable cost–benefit profile compared to MDCT due to a higher spatial resolution of CBCT with lower radiation dose exposure, providing excellent image quality for bone visualization^19,21,32,33^.

With regard to the assessment of the integrity of the subchondral bone plate, there is a lack of studies demonstrating the benefit of CBCT in larger human cohorts and/or long-term follow-up. In a previous study in race horses, the superior sensitivity of CBCT compared to MRI for detecting fissure fractures of metacarpal/tarsal parasagittal groove and proximal phalanx was demonstrated^34^. Novel MRI sequences are currently introduced in experimental settings using chemical shift sequences or so called “black bone sequences”. These additional MRI data sets offer the potential to provide relevant information regarding the subchondral bone plate evaluation. Indeed, MRI is currently more widely available among different types of hospitals, while CBCT modality is predominantly used in few specialized hospitals. However, the availability of MRI slots in clinical routine is very limited. Due to the shorter scan time of CBCT compared to MRI, CBCT could be relatively more cost effective for certain musculoskeletal conditions, although there are no corresponding studies to date.

Another limitation of our study is that the MRI and CBCT scans were not performed on the same day. The scans were acquired as part of the routine workflow and therefore had a temporal offset. Importantly, all patients who differed in grading between the two modalities showed short intervals between MRI and CBCT imaging. Therefore, it seems unlikely that temporal progression of osseous changes caused the discrepancy in grading. Notably, previously undiagnosed osteoarthritic changes can also promote the development of subchondral lesions. Based on the sudden atraumatic onset of pain with typical clinical and radiological findings, the origin of the osseous lesion in our study cohort was interpreted as a consequence of a subchondral insufficiency fracture. According to the inclusion criteria, MRI did not show osteoarthritic changes in any of the cases. It should also be noted that the MRI examinations were carried out externally in advance. Accordingly, the MRI protocols of the individual patients were not identical. Imaging artifacts can become a relevant confounding factor for the evaluation of MRI images of different protocols. However, our ROIs in the femoral condyles were placed close to the center of each sequence and therefore without any influence, such as chemical shift artifacts. In addition, none of the patients had undergone previous knee surgery, so no artifacts due to foreign bodies could be detected.

In order to strengthen the validity of our study with a limited sample size, we also determined the degree of agreement between the severity gradings and the effect sizes in the assessment of the lesion variables. We found a disagreement in the intermodal grading score and a large effect size for the analysis of the lesion sizes. Last, our study lacks follow-up MRI and CBCT scans as well as longitudinal assessments of clinical outcomes. Further studies should include longitudinal data that assess true diagnostic efficacy by evaluating treatment decisions based on additional CBCT lesion grading and associated clinical outcomes. Nelson et al. have already described the potential of CBCT bone texture assessment for subchondral bone response as a clinically relevant predictive tool for long-term OA assessment^35^.

## Conclusion

CBCT represents our imaging modality of choice for diagnosing subchondral fractures by offering advantages compared to MRI, especially in the assessment of subchondral bone plate integrity and lesion size. These results are clinically relevant because precise assessment of subchondral parameters often determines conservative or surgical treatment. MRI is still the gold standard for the detection of early stages of bone marrow edema (Supplementary Figures).

### Supplementary Information

Supplementary Figures.

## Data Availability

The datasets generated and analyzed during the current study are not publicly available due restrictions of the local ethics committee but are available from the corresponding author on reasonable request.
