# Supplementary figures and images for: Advantages of cone beam computed tomography for evaluation of subchondral insufficiency fractures of the knee compared to MRI
Source: Sci Rep. 2024 Jul 3;14:15278. doi: 10.1038/s41598-024-64591-7 (PMC11222521; doi:10.1038/s41598-024-64591-7)

Figure 1

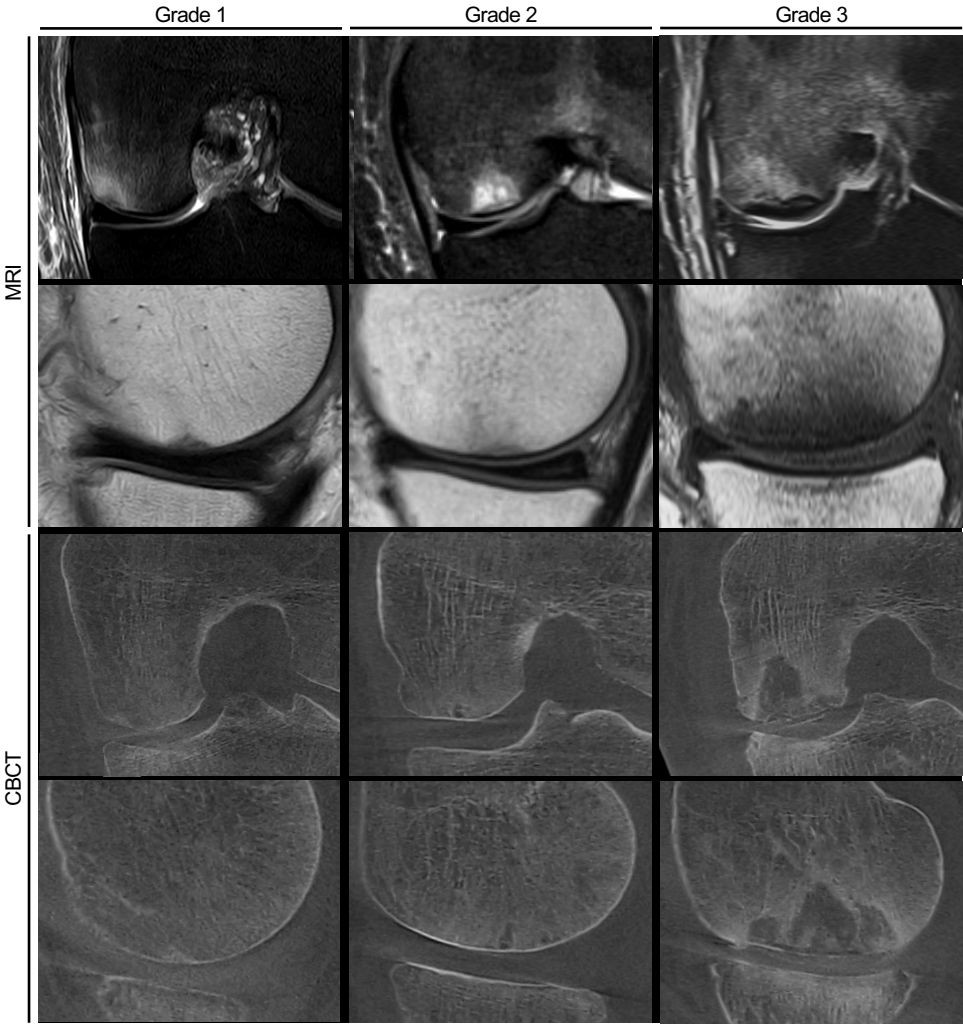

Figure 2

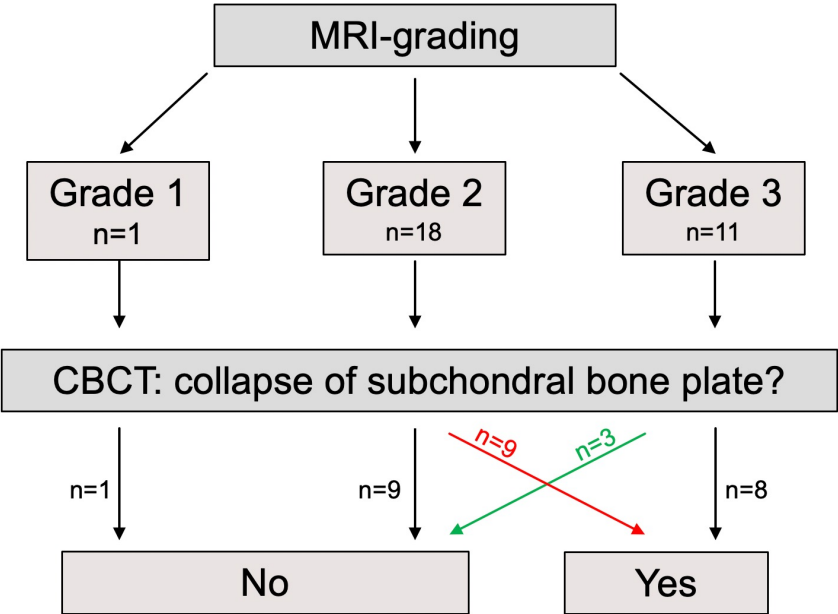

Figure 3

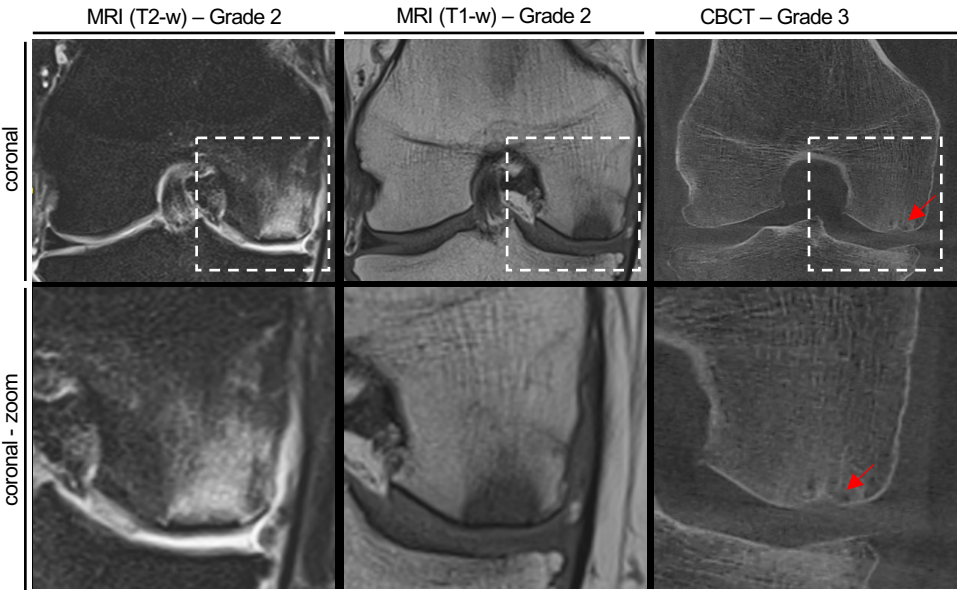

Figure 4

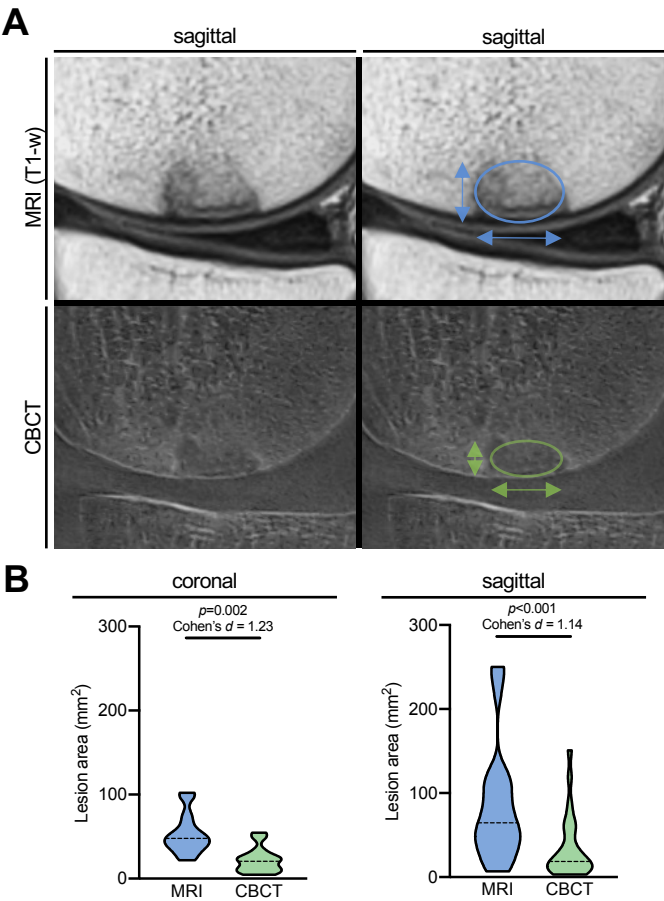

Supplement: Supplementary file 1 — Supplementary Figures. [file 41598_2024_64591_MOESM1_ESM.pdf]
